# Unlocking Responsive and Unresponsive Signatures: A Transfer Learning Approach for Automated Classification in Cutaneous Leishmaniasis Lesions

**DOI:** 10.1155/tbed/5018632

**Published:** 2025-01-21

**Authors:** Mehdi Bamorovat, Iraj Sharifi, Amirhossein Tahmouresi, Setareh Agha Kuchak Afshari, Esmat Rashedi

**Affiliations:** ^1^Leishmaniasis Research Center, Kerman University of Medical Sciences, Kerman, Iran; ^2^Machine Learning and Modelling Expert, Kerman University of Medical Science, Kerman, Iran; ^3^Medical Mycology and Bacteriology Research Center, Kerman University of Medical Sciences, Kerman, Iran; ^4^Faculty of Electrical and Computer Engineering, Graduate University of Advanced Technology, Kerman, Iran

## Abstract

Cutaneous leishmaniasis (CL) remains a significant global public health disease, with the critical distinction and exact detection between responsive and unresponsive cases dictating treatment strategies and patient outcomes. However, image-based methods for differentiating these groups are unexplored. This study addresses this gap by developing a deep learning (DL) model utilizing transfer learning to automatically identify responses in CL lesions. A dataset of 102 lesion images (51 per class; equally distributed across train, test, and validation sets) is employed. The DenseNet161, VGG16, and ResNet18 networks, pretrained on a massive image dataset, are fine-tuned for our specific task. The models achieved an accuracy of 76.47%, 73.53%, and 55.88% on the test data, respectively, with a sensitivity of 80%, 75%, and 100% and specificity of 73.68%, 72.22%, and 53.12%, individually. Transfer learning successfully addressed the limited sample size challenge, demonstrating the models' potential for real-world application. This work underscores the significance of automated response detection in CL, paving the way for treatment and improved patient outcomes. While acknowledging limitations like the sample size, the need for collaborative efforts is emphasized to expand datasets and further refine the model. This approach stands as a beacon of hope in the contest against CL, illuminating the path toward a future where data-driven diagnostics guide effective treatment and alleviate the suffering of countless patients. Moreover, the study could be a turning point in eliminating this important global public health and widespread disease.

## 1. Introduction

Leishmaniasis is endemic in many countries and has targeted poor societies in tropical areas [1–3]. Depending on the parasite species and region, it shows different clinical forms [4, 5]. Among them, cutaneous leishmaniasis (CL) is the most predominant form [6–8]. Despite its global extent, affecting roughly 12 million people and causing 1–2 million new cases annually. CL is considered to be one of the most neglected tropical diseases (NTDs) [3, 8, 9]. This means it primarily afflicts impoverished communities in both tropical and subtropical regions, where resources for research, diagnosis, and treatment are scarce. This neglect fuels a vicious cycle: limited awareness leads to delayed diagnosis and inadequate treatment, perpetuating transmission, and sustaining poverty. CL is not just a skin disease; it can cause disfiguring scars, impair social interactions, and hinder economic opportunities. Recognizing it as an NTD and mobilizing resources for research, prevention, and accessible treatment is crucial to breaking this cycle and alleviating the suffering of millions [10–13].

CL imitates a range of disease conditions, infections, and noninfectious disorders comprising viral, fungal, parasitic, and bacterial infections, lupus vulgaris, tuberculosis, sporotrichosis, zoster, herpes-like and wart viruses, mycobacterial ulcers, cutaneous diseases, myiasis, tropical ulcers, ecthyma, foreign-body granuloma, acute furunculosis, and carcinoma of the skin [14–16]. Accurate knowledge of these demonstrations and confirmation of the disease is very important for selecting the appropriate treatment in endemic areas [4, 14, 15, 17, 18].

The complex landscape of CL is further muddied and puzzled by the presence of responsive and unresponsive cases [5]. While some patients heal effectively with standard treatments, others suffer chronic and unresponsive lesions that resist therapy and cause immense personal and economic hardship [5]. Accurately differentiating between these groups is critical for optimizing care. Responsive cases can be cured swiftly with minimal intervention, preventing long-term scarring and disability. On the other hand, misidentifying an unresponsive case could lead to ineffective treatment regimens, delaying healing, and potentially necessitating more aggressive and expensive interventions. Therefore, developing precise methods to identify responsive and unresponsive patients stands as a pivotal step in efficiently managing this neglected disease [13].

The tide is turning in the fight against neglected diseases like CL. Advancements in machine learning and artificial intelligence (AI) offer unprecedented medical diagnosis breakthroughs, and CL is no exception [12]. These data-driven tools can sift through vast amounts of complex medical data, including images, genomic information, and clinical records, uncovering subtle patterns and correlations that human eyes might miss [5, 19]. This opens up the possibility of developing highly accurate and noninvasive methods for identifying responsive and unresponsive CL cases, leading to a revolution in diagnosis and treatment. Imagine AI algorithms seamlessly analyzing lesion images, pinpointing biomarkers from blood samples, or even predicting treatment response before it unfolds. This could pave the way for personalized therapeutic strategies, tailoring interventions to individual needs and drastically improving patient outcomes. The dawn of AI-powered diagnostics holds immense promise for tackling the challenges of CL and potentially transforming the trajectory of this neglected disease. There are few studies regarding AI to diagnose infectious diseases like CL. Bamorovat et al. [20] showed a novel prognostic and diagnostic approach for CL classifying comprising responsive and refractory cases, they demonstrated the possible automatic proof of identity by ML algorithms. Noureldeen, Masoud, and Almakhzoom [21] displayed an innovative diagnostic model of CL detection and identification using the ML method. A novel procedure in this regard is to evaluate images of CL relative to other cutaneous lesions due to infectious and noninfectious diseases. They applied AlexNet to assist in the CL diagnosis.

In this vein, we propose the development of a novel and automated approach for classifying responsive and unresponsive cases of CL. This approach harnesses the power of deep learning (DL) through a transfer learning technique. By leveraging the pretrained capabilities of robust networks like DenseNet161, ResNet18, and VGG16, we aim to extract intricate features from lesion images that might escape traditional analysis. This paves the way for a data-driven and noninvasive method capable of accurately distinguishing between patients who will likely respond well to standard treatment and those who may require more tailored or aggressive interventions.

## 2. Materials and Methods

### 2.1. Study Design

A case-control analysis was managed between February 2023 and January 2024 in major foci of ACL in Bam and Kerman districts, southeastern Iran. This investigation was completed in two collections with different results in terms of therapy outcomes. One group (control) responded thoroughly to meglumine antimoniate (Glucantime, MA) and another group (case) was refractory.

### 2.2. Ethical Consideration

The process of ethical consideration was approved by the Ethical Committees of Kerman University of Medical Sciences (Ethic number IR. KMU. REC.1402.479, contract no. 402000993). Originally, the purpose of the study, measures, and probable benefits were described to the subjects. CL cases contributed voluntarily and written informed consent was taken from all patients. Also, “in preference” of the children, parents completed the consent. The data source was initiated from the confirmed unresponsive and responsive cases, which were already documented in the electronic database and backup record-keeping book in the referral clinic.

### 2.3. Case Ascertainment

**Figure 1:**
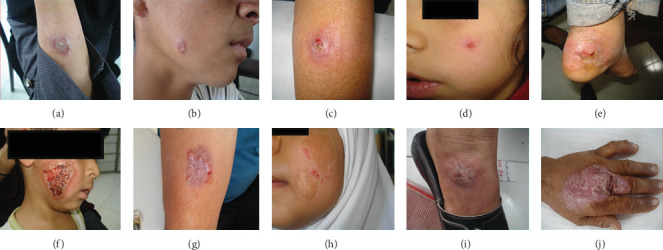
Representative images of skin lesions of responsive (A–E) and unresponsive (F–J) patients with cutaneous leishmaniasis (CL) from the Kerman and Bam districts [5, 22–24].

Subjects were randomly selected from the main ACL focal points (Bam and Kerman) from unresponsive and responsive. The unresponsive patient has not been healed and continued with an active lesion, despite receiving three cycles of intralesional MA (20 mg/kg/weekly) with biweekly liquid nitrogen cryotherapy for 12 weeks or systemic MA alone (20 mg/kg/daily for 3 weeks) and at least 1 year has passed since the appearance of their lesions. The responsive patient is one whose skin lesion has been cured by one treatment course with intramuscular administration of MA alone or intralesional MA together with biweekly cryotherapy as mentioned above with no CL relapse next 6 months of the follow-up judgment.  Figure 1 shows cutaneous lesions of responsive and unresponsive cases with CL. Totally, clear and high-quality images of lesions with proper distance were taken from 102 patients (51 images per group) that were included in the study. We took pictures of the patient's lesions with a Sony alpha 7 R III-ICLE-R7M2 camera (Sony Corp, Tokyo, Japan).

### 2.4. Pretrained DL Network

In the context of CL lesions, “responsive” and “unresponsive” signatures refer to the observable characteristics of lesions that indicate how well they respond to treatment.

• Responsive signatures: These are lesions that exhibit positive changes or improvements following treatment. This includes a reduction in size, decreased inflammation, and healing of the affected tissue. The criteria for classifying a lesion as responsive are based on clinical observations such as the rate of lesion shrinkage, reduction in erythema, and the overall appearance of healing.
• Unresponsive signatures: In contrast, unresponsive signatures refer to lesions that do not show significant improvement or may even worsen despite treatment. These lesions may remain the same in size or become more inflamed or ulcerated. Criteria for unresponsiveness include a lack of change in lesion dimensions, persistent or increased inflammation, and lack of healing over a set period.

We employ a combination of clinical evaluation and imaging techniques to classify lesions as responsive or unresponsive. Key criteria include:

1. Size measurement: Regular monitoring of lesion dimensions is performed. A decrease in size over time generally indicates responsiveness.
2. Inflammation assessment: The degree of redness and swelling is observed. A reduction in these signs suggests a positive response to treatment.
3. Tissue healing: The extent of re-epithelialization or scarring is assessed. Healing tissue indicates responsiveness, whereas persistent ulceration or necrosis suggests unresponsiveness.

These criteria are incorporated into our automated classification approach, allowing us to distinguish between responsive and unresponsive lesions using the features extracted from the images. The automated system leverages these defined criteria to classify the lesions based on their observable signatures in the collected images.

**Figure 2:**
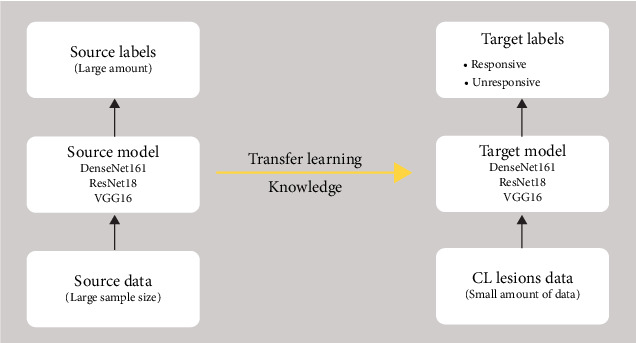
The transfer learning obtaining knowledge workflow for cutaneous leishmaniasis (CL) classification problem.

At the heart of our automated detection approach lies the powerful technique of transfer learning, which is shown in Figure 2 for this classification problem [25]. This approach leverages the knowledge gleaned from the previously trained DL networks—a seasoned veteran if you will—and applies it to a new and related task [26]. In our case, the models are the DenseNet161, ResNet18, and VGG16 networks, these convolutional neural networks (CNNs) are pretrained on a massive dataset of images [27]. It has already learned valuable lessons about extracting intricate features from visual data, which we can capitalize on for our CL challenge.

Imagine transfer learning as a bridge over a vast chasm [28]. On one side lies the vast terrain of image recognition models have conquered. On the other lies our specific task of distinguishing responsive and unresponsive CL lesions. Transfer learning allows us to leverage the robust features and patterns these networks have already identified, adapting them to navigate the nuances of skin lesion analysis.

Here's how we will employ this strategy:

Fine-tuning: We would not discard the expertise of these networks. Instead, we will carefully adjust their final layers, specifically those responsible for classification, to focus on the subtle differences between responsive and unresponsive CL lesions.

Feature extraction: By analyzing the activations of the networks' pretrained layers, we can glean valuable insights into the features it deems important for image recognition. These learned features become the starting point for our models, providing a head start in understanding the intricacies of CL lesion images.

Reduced training data needs: Transfer learning allows us to train our models effectively even with limited datasets of CL lesion images. This is crucial in the context of NTDs like CL, where data resources are often scarce.

By harnessing the transfer learning bridge, we hope to accelerate the development of our model, improve its accuracy, and ultimately provide a novel tool for the efficient and potentially life-changing diagnosis of CL.

As you can see, the architecture of this classification problem via transfer learning is shown in Figure 2. The number of convolutional layers and filters increases progressively as we move through the blocks, allowing the networks to extract increasingly complex features from the input image.

Here's a breakdown of the component's networks:

1. Input: The network takes an image as input typically resized to 224 × 224 pixels.
2. Convolutional layers: These layers extract features from the image by convolving them with small filters. Each filter detects specific patterns in the image.
3. To enhance the diversity and robustness of our dataset, we applied data augmentation techniques, including rotation adjustments.
4. Activation functions: Introduced nonlinearity into the network, preventing it from just learning linear relationships between the input and output.
5. Max pooling layers: Down-sample the feature maps, reducing the image size and computational complexity while retaining important information.
6. Fully connected layers: These layers take the flattened feature maps from the convolutional layers and use them to make predictions about the image.
7. Output: The network finally outputs a probability distribution over the possible classes for the input image.

**Figure 3:**
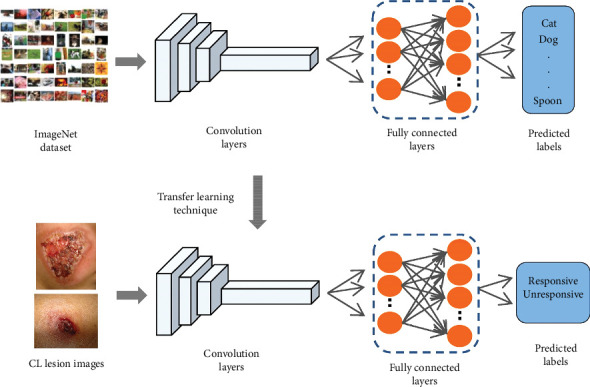
The architecture of the transfer learning technique in the classification of responsive and unresponsive cases.

In our context, we will utilize the pretrained features extracted by the convolutional layers of the three mentioned models and fine-tune the fully connected layers to distinguish between responsive and unresponsive CL lesions. In addition, we divided our data into 70% for training and validation, and the remaining 30% to test the models, and Adam optimizer is allocated to optimizing the weight and biases of the networks. The architecture of our approach is shown in Figure 3.

## 3. Results

Building a robust model requires a solid foundation, and our data plays a crucial role in this endeavor. Our dataset comprises three parts: training, testing, and validation, each containing an equal representation of both responsive and unresponsive CL cases. While we have 51 images per class, ensuring balanced representation, the limited number of unresponsive patient images presents a significant challenge.

This scarcity poses a hurdle for training a model from scratch, as insufficient data can lead to overfitting and impede generalizability. Fortunately, this is where the power of transfer learning shines. By leveraging the pretrained capabilities of the DenseNet161, ResNet18, and VGG16 networks, we can circumvent the data limitation. These three networks honed on a vast dataset of diverse images and have already learned valuable feature representations that can be adapted to our specific task of classifying CL lesions. This allows us to train our model effectively even with our relatively small dataset, making the most of the available data and increasing the likelihood of building a model that generalizes well to unseen images.

In essence, the limited availability of unresponsive patient images makes transfer learning a strategic choice. It acts as a bridge, utilizing the pretrained knowledge to navigate the limited data landscape and pave the way for a robust and accurate model capable of distinguishing responsive and unresponsive CL cases.

**Figure 4:**
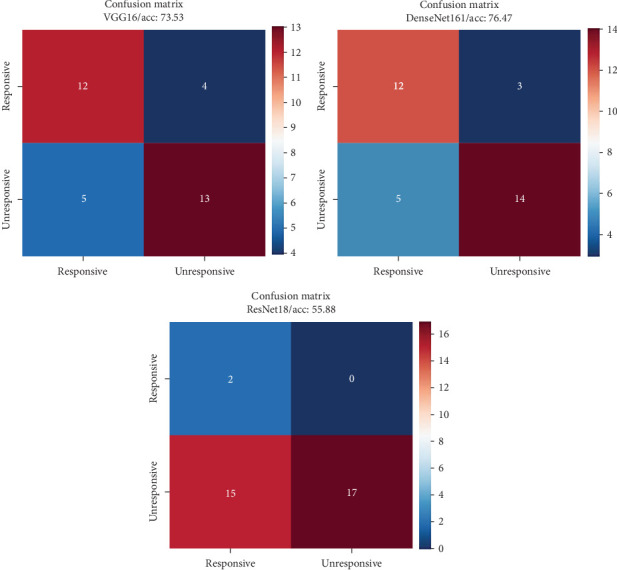
Confusion matrices with accuracies for VGG16, DenseNet161, and ResNet18 on the test data.

Evaluating our models' performance on the test data reveals promising results.  Table 1 summarizes the key classification metrics, with an overall accuracy of 76.47% for DenseNet161 demonstrating the model's ability to accurately distinguish between responsive and unresponsive CL cases. The sensitivity (recall) of 80% and specificity of 73.68% for this network showcase its effectiveness in identifying both patient groups, and in Figure 4, the confusion matrices for models are prepared for better classification visualization. The classification metrics could be calculated for Equations 1 to 7.

1. True positive (TP) rate (sensitivity) = $\frac{\text{TP}}{\text{TP}+\text{FN}}$.
2. True negative (TN) rate (specificity) = $\frac{\text{TN}}{\text{TN}+\text{FP}}$.
3. Positive predictive value (precision) = $\frac{\text{TP}}{\text{TP}+\text{FP}}$.
4. Sensitivity = recall × 100.
5. Test accuracy = $\frac{\text{TP}+\text{TN}}{\text{TP}+\text{TN}+\text{FP}+\text{FN}}$.
6. F1 score = $\frac{2\text{TP}}{2\text{TP}+\text{FP}+\text{FN}}$.
7. Error rate = 1 − test accuracy.

**Figure 5:**
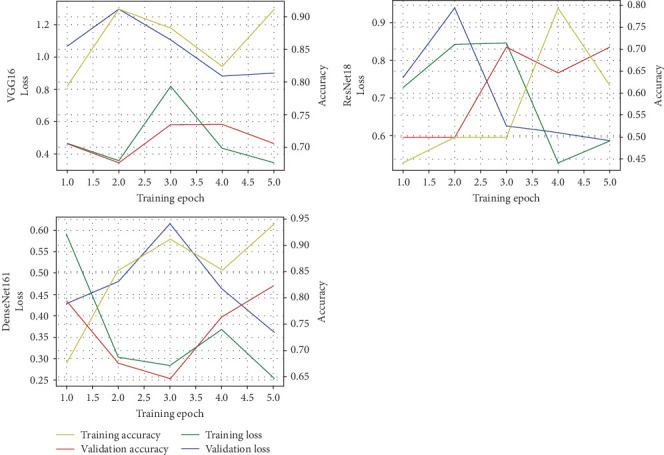
Training and validation accuracy and loss over epochs for three networks.

Figure 5 indicates the training and validation accuracy curves over epochs, indicating effective learning without overfitting. This conception also suggests the superior model's ability to generalize well to unseen data.

**Figure 6:**
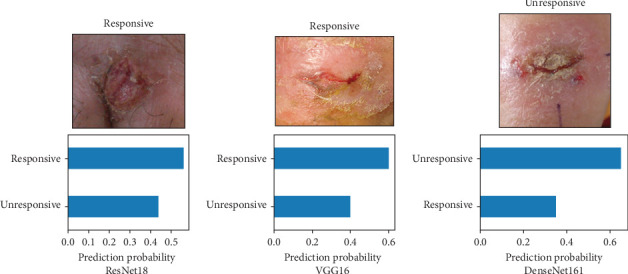
The models' predictions on the random image of the test set.

Moving beyond metrics, Figure 6 beautifully exemplifes the model's real-world potential. Here, it accurately predicts the unresponsive class for two random test images, highlighting its promise for clinical application in aiding diagnosis and guiding treatment decisions. These encouraging results pave the way for further fine-tuning and validation as we move toward real-world implementation of this AI-powered tool for identifying responsive and unresponsive CL cases.

## 4. Discussion

CL is a treatable disease; though due to several factors, refractory cases to treatment are inevitable. A significant and major problematic issue regarding the treatment schedule is unresponsiveness, aside from parasitic diseases such as leishmaniasis likewise in other infectious diseases as well [5, 18, 29]. In this study, DenseNet161, VGG16, and ResNet18 networks, pretrained on a considerable image dataset, were fine-tuned for our particular task. The networks succeeded with an accuracy of 76.47%, 73.53%, and 55.88% on the test data, respectively, with a sensitivity of 80%, 75%, and 100% and specificity of 73.68%, 72.22%, and 53.12%, individually. In the intricate world of CL, accurately distinguishing the battle lines between responsive and unresponsive cases is paramount for tailoring effective treatment strategies [5]. This distinction rises above a mere diagnosis; it determines the course of healing, the potential for scarring, and ultimately, the patient's quality of life. Yet, in the expanding field of image-based research, this crucial differentiator has remained largely unexplored. Enter our study, lighting illuminating the path towards automated response detection, a pivotal step in revolutionizing CL management. However, we are at the beginning and finding the best model for diagnosis requires additional studies and a larger sample size.

Currently, there have been developments and successes in several fields of medical sciences. The connection among diverse fields of science has also created a great involvement in this advancement. Early detection and precise prediction are fundamentals for choosing the suitable treatment regimen and effective means for the management of CL unresponsive cases. Advances in diagnostic procedures with high values of specificity and sensitivity are useful avenues for radical disease control.

DL procedures can benefit the diagnosis of skin diseases through the analysis of lesion images. They obtained outlines from images and created estimates from data sources. This approach is a significant step in assisting a precise diagnosis, enabling and supporting clinical decision-making [30–32]. All procedures, medical involvements, and diagnostic assessments are not accurate and need evaluation before introduction into the healthcare system. Hence, the accuracy of diagnosis is vital in differentiating between true and false cases [33]. This parameter can be measured using an accuracy assessment related to sensitivity and specificity. The main aims of diagnostic methods must be to prevent the severity of diseases, diminish misery, and improve the effectual health of patients. In this regard, novel methods can develop the diagnosis, encourage patients, and help physicians provide proper treatment [34–38].

Building a DL model for this specific task holds immense significance. Unlike conventional methods, our AI-powered approach delves into the intricate visual language of skin lesions, detecting subtle patterns and distinctions that may elude even the most seasoned clinician. This unveils a new landscape of possibilities, where responsive cases can be swiftly ushered towards optimal treatment pathways, while unresponsive ones receive the targeted interventions they desperately need. The potential to minimize unnecessary cycles of ineffective therapy, mitigate scarring, and improve patient outcomes is truly game-changing.

However, this journey does not exist in a vacuum. We readily acknowledge the limitations inherent in our study. The relatively small sample size, an ever-present challenge in NTDs like CL, could introduce biases and limit the model's generalizability. Yet, it is precisely in these resource-constrained landscapes that the brilliance of transfer learning shines. By leveraging the pretrained wisdom of established DL architectures like VGG16, we could bridge the data gap, extracting critical insights and building a robust model even with limited resources. This strategy serves as a point of hope, demonstrating that cutting-edge AI can blossom even in the face of scarcity.

Despite these strides, the need for larger and diverse datasets remains undeniable. Each additional image acts as a brushstroke, refining the portrait of CL response and enriching the model's understanding. Collaborations with research institutions and clinicians across endemic regions become crucial in this endeavor. As the dataset expands, so too will the model's proficiency, solidifying its position as a reliable tool in the real-world clinical setting.

In a study, Choy et al. [39] examined the most widespread cutaneous lesions such as acne, psoriasis, eczema, rosacea, and urticarial using DL algorithms and predicted the precision rate for detecting these lesions to be about 90%. In another study, the authors advanced a procedure using different DL algorithms to identify noncontagious skin lesions [40]. Their findings demonstrated that MobileNet V2 had more correctness on lightweight computing programs. AlSuwaida [41] assessed the presentation of VGG16, EfficientNet, InceptionV3, MobileNet, NasNet, and ResNet50 for common dermatological lesions including eczema, atopic dermatitis, and psoriasis in the Middle East. The results showed that the MobileNet had the most accuracy (95.7%).

For the detection of actinic keratosis, Li, Desrosiers, and Liu [42] demonstrated the efficiency of various CNNs with a precision of about 92%. Also, Bisla et al. [43] used DL and compared it to the common baseline methods for the classification of melanoma. Noureldeen, Masoud, and Almakhzoom [21] applied the YOLOv5 model for detecting CL and found an average accuracy of 70%. A study by Thieme et al. [44] performed a CNN to classify the features of skin lesions due to monkeypox (sensitivity of about 90%). Leal et al. [45] obtained a mean accuracy of 95.04% by applying AlexNet in detecting images of CL manifestations. They declared that CL differentiation by AlexNet is likely to assist clinicians in appropriate treatment and essential care. Finally, Clésio Gonçalves demonstrated that trained DL models with microscopic slide imaging can accurately assist in the exact detection of VL [46].

Our work is not merely a technical achievement; it is a clarion call for collective action. We stand at the precipice of a paradigm shift in CL management; one where personalized, data-driven treatment paves the way for a brighter future for patients. By acknowledging limitations, embracing collaboration, and tirelessly pursuing knowledge, we can illuminate the path toward overcoming the shadow of leishmaniasis and ensuring equitable access to life-changing care.

The high accuracy of DenseNet161 and its better performance in classifying images in our approach, coupled with its ability to navigate limited data challenges, demonstrate its real-world potential. Imagine a future where clinicians, armed with this AI-powered tool, can swiftly discern responses, directing patients towards optimal treatment paths. This could not only improve healing rates and minimize scarring, but also alleviate the immense emotional and economic burden borne by countless patients.

Yet, our journey is far from over. We readily acknowledge the need for further refinement and validation through expanded datasets and collaborative research efforts. As the landscape of data enriches, so too will the models' accuracy and generalizability, solidifying their role as a reliable clinical tool. Furthermore, integrating this technology into existing healthcare infrastructure and fostering clinician training will be crucial for seamless integration into patient care pathways.

Ultimately, our work stands as a testament to the transformative power of AI in tackling NTDs like CL. It embodies a hopeful vision of a future where data-driven diagnostics guide the way toward precision medicine, ensuring equitable access to effective treatment for all. This is not merely a technological advance; it is a beacon of hope, ushering in a dawn of precision in the fight against CL and beyond.

This study had some limitations. A larger sample size was needed to achieve the best results. Also, this work is a preliminary study that needs to be strengthened from various aspects. In this regard, additional studies are needed to test more models and networks to achieve the highest accuracy. Noninvasive, quick, inexpensive, and easy sampling are the strengths of the study. Another strength was that the sampling was done in a well-known focus of CL, which has a functional registry system, and the various forms of the disease are well distinguished.

## 5. Conclusion

Our study illuminates a novel approach to the fight against CL. By harnessing the power of deep and transfer learning, we have developed automated models capable of accurately identifying responsive and unresponsive cases with CL based on lesion images, and the DenseNet161 network has been superior in our classifying task. This marks a significant shift in the paradigm of CL diagnosis, paving the way for proper treatment strategies and improved patient outcomes that finally can help control the disease. This action could be an initial step toward the development of an advanced model that can enable more sensitive diagnosis and treatment of this neglected disease in the future.

## Figures and Tables

**Table 1:** Classification metrics on test data for three networks.

Metric	VGG16	ResNet18	DenseNet161
True positive (TP)	12	2	12
True negative (TN)	13	17	14
False positive (FP)	5	15	5
False negative (FN)	4	0	3
Precision	0.71	0.12	0.71
Recall	0.75	1	0.80
Sensitivity (%)	75.00	100	80
Specificity (%)	72.22	53.12	73.68
F1 score	0.73	0.21	0.75
Test accuracy (%)	73.53	55.88	76.47
Error rate (%)	26.47	44.12	23.53

## Data Availability

The data that support the findings of this study are available from the corresponding author upon reasonable request.
